# EMS-induced mutagenesis in Choy sum (*Brassica chinensis* var. *parachinensis*) and selection for low light tolerance using abiotic stress indices

**DOI:** 10.1186/s12870-023-04570-8

**Published:** 2023-11-21

**Authors:** Subramanian Deivanai, Benny Jian Rong Sng, Kien Van Vu, Thankaraj Salammal Maria Shibu, In-Cheol Jang, Srinivasan Ramachandran

**Affiliations:** 1 School of Applied Sciences, Republic Polytechnic, 9 Woodlands Ave 9, Singapore, 738964 Singapore; 2 Temasek Life Sciences Laboratory Limited, Research Link, National University Singapore, Buona Vista, Singapore, 117604 Singapore

**Keywords:** Choy sum, Controlled environment agriculture, EMS mutagenesis, Abiotic stress tolerance indices, Shade tolerance

## Abstract

**Background:**

Choy Sum (*Brassica rapa* ssp. *chinensis* var. *parachinensis*), grown in a controlled environment, is vulnerable to changes in indoor light quality and displays distinct photo-morphogenesis responses. The scarcity of Choy Sum germplasm for indoor cultivation necessitates the development of new cultivars. Hence, this study attempted to develop mutants through chemical mutagenesis and select low-light-tolerant mutants by using abiotic stress tolerance indices.

**Results:**

A mutant population of Choy Sum created using 1.5% ethyl methane sulfonate (EMS) at 4 h was manually pollinated to obtain the M2 generation. 154 mutants with reduced hypocotyl length were initially isolated from 3600 M2 seedlings screened under low light (R: FR = 0.5). Five mutants that showed reduced plant height at mature stages were selected and screened directly for shade tolerance in the M3 generation. Principal component analysis based on phenotypic data distinguished the M3 mutants from the wild type. Abiotic stress tolerance indices such as relative stress index (RSI), stress tolerance index (STI), geometric mean productivity (GMP), yield stability index (YSI), and stress resistance index (SRI) showed significant (*P* < 0.05), and positive associations with leaf yield under shade. M3-12–2 was selected as a shade-tolerant mutant based on high values of STI, YSI, and SRI with low values for tolerance (TOL) and stress susceptibility index (SSI).

**Conclusions:**

The results demonstrate that mutation breeding can be used to create dominant mutants in Choy Sum. Furthermore, we show that screening for low light and selection based on abiotic tolerance indices allowed the identification of mutants with high resilience under shade. This method should apply to developing new cultivars in other crop plants that can be suitable for controlled environments with stable yield performance.

**Supplementary Information:**

The online version contains supplementary material available at 10.1186/s12870-023-04570-8.

## Introduction

Choy Sum *(Brassica rapa* ssp. *chinensis* var. *parachinensis*) is one of the popular Asian green leafy vegetables, originally from southern China and cultivated in most parts of Southeast Asia. It is grown for its edible leaves and flowering stalks, which are rich in phytochemicals, vitamins, minerals, and dietary fibers and are commonly used in Chinese cuisine. The leaves are good sources of glucosinolates, and anthocyanins are known to have anti-aging, antioxidant, and anti-cancer properties [1, 2]. Choy Sum’s short life cycle and rapid growth allows it to grow in a climate-controlled environment and provide a consistent supply of leafy greens all year. Controlled Environment Agriculture (CEA) technology has been widely practiced in countries like Australia, China, Europe, Israel, Japan, Singapore, and South Korea [3, 4]. In CEA, light-emitting diodes (LEDs) are the major contributing factor in influencing plant growth and are used in combinations of white, red, and blue lights. However, the crops selected for CEA were sensitive to different spectral compositions of light and displayed distinct photo-morphogenesis responses, such as elongated stems, vertically oriented leaves, reduced branching, a decreased photo-assimilation rate, and lower yield [5–9]. This complex phenomenon is referred to as “shade avoidance response” (SAR), which is detrimental to crop plants as it decreases resource allocation vital for growth and development [10]. Studies have suggested that the use of reduced SAR is probably advantageous to the growers to maximize the yield in CEA [7, 11, 12]. In such situations, the appropriate selection of cultivars with canopy shade tolerance is of paramount importance in achieving the expected crop yield. However, the lack of a suitable cultivar may be a major barrier to the expansion of the CEA; hence, it is necessary to develop Choy Sum cultivars adaptable to controlled environments.

Induced mutagenesis generates heritable variations, which are easier to screen for specific mutations in a population [13–15]. Several superior plant varieties with high grain yield, early maturity, lodging resistance, etc., were developed globally by this method [16, 17]. Ethyl methane sulfonate (EMS) is a widely used chemical mutagen to induce genetic variations in crop plants and has gained popularity due to its effectiveness and ease of handling. However, the usefulness of the mutagen relies on dose optimization to achieve the least possible unintended damage to the crop [18–20]. Furthermore, identification of a novel target mutant begins with screening and selection of useful variants from a large, mutated population, followed by confirming the putative mutants by re-evaluating them in a controlled environment with replications [21–23]. Visual screening on morphological traits like disease resistance, flowering earliness, plant height, adaptation to the soil, climate, growing period, etc. is commonly practiced in the M2 generation [22, 24]. Thinner leaves with reduced apical dominance, short internodal length, high branching frequency with low elongation response, and higher chlorophyll content per leaf area were found to impact the fitness of individuals under low light conditions in response to reduced shade avoidance [25–29]. In a study, Li et al. [30] selected a putative shade-tolerant mutant of perennial ryegrass by identifying a de-etiolated phenotype with short coleoptiles, emergent true leaves, and reduced seedling height. They demonstrated that the de-etiolated phenotype was favored by the insensitivity of seedlings to low light. Furthermore, semi-dwarf or dwarf crop plants of rice, barley, cotton, tomato, potato, and tobacco were capable of suppressing shade avoidance and improving crop yield in certain production systems [31–36].

Several morphological and physiological parameters such as plant growth, leaf area, rate of photosynthesis, leaf moisture content, yield components, etc., have been proposed for evaluating stress tolerance in plants. However, it is crucial to select the most sensitive parameters to be measured to improve the stability of newly developed genotypes. Besides, the selection of stress-adaptive attributes is complex and often interrelated with various physiological and biochemical adaptations to low light. The most common adaptation of many shade-tolerant plants is their capacity to maintain adequate photosynthesis and regulate enzyme activities, especially ion sequestration, metabolic and osmotic adjustment, and antioxidative defense [37–40]. In general, shade-adapted plants are characterized by the very low photosynthetic capacity that is inevitably associated with the production of reactive oxygen species (ROS) [41, 42]. Quantitative measures of chlorophyll and enzyme activities, e.g., peroxidase (POD), catalase (CAT), ascorbate peroxidase (APX), and superoxide dismutase (SOD), were recognized as physiological indices and used as screening tools for the identification of stress-tolerant plant species [43, 44]. Additionally, several numerical descriptors of tolerance, such as the measure of tolerance index (TOL), stress susceptibility index (SSI), stress tolerance index (STI), mean productivity (MP), geometric mean productivity index (GMP), yield index (YI), stress susceptibility index (SSPI), and abiotic tolerance index (ATI), have also been suggested for the selection of genotypes based on their yield performance in natural and stress conditions where TOL and MP represent the difference in yield and mean yield under stress and non-stress conditions [45]. The SSI estimates the yield stability that accounts for both potential and actual yields in variable environments [46]. The STI and GMP identify highly productive genotypes in both stress and non-stress conditions [47]. The YI and yield stability index (YSI) evaluate the stability of genotypes in both stress and non-stress conditions [48, 49]. The SSI and ATI recognize relatively tolerant and non-tolerant genotypes [50].

The polymerase chain reaction-restriction fragment length polymorphism (PCR–RFLP) of phytochrome genes was used to identify cultivars. The Phytochromes, which mediate photomorphogenic responses in the adaptation of growth and development in angiosperms, are of three major types namely PHYA, PHYB, and PHYC. According to Mathews [51] and Song et al., [52], PHYB is stable while PHYA is low in light, and both together mediate the SAR in conditions of extended shade and enable the plant to become shade tolerant. Here we.

describe the establishment of the EMS mutant population in Choy Sum, as well as the use of low light intensity to screen M2 seedlings for shade tolerance. We isolated putative shade-tolerant M3 mutants and evaluated the morphological and physiological parameters, comparing the isolated mutant’s response with the wild type in both light and shade conditions. To investigate the possibility of identifying stable tolerant M3 mutants with reduced shade avoidance responses, ideal for a controlled environment, stress tolerance indices were estimated and analyzed.

## Materials and methods

### Plant material and EMS dose optimization

A greenhouse study was carried out at Temasek life sciences laboratory (Singapore) and the study did not involve endangered or protected species.

A cultivated Choy Sum (*Brassica rapa* ssp. *chinensis* var. *parachinensis*) seed “Hong Kong Choy Sum’ obtained from Ban Lee Huat Seed Pte Ltd., Singapore, was used in the study. The seeds (50 seeds per treatment) were pre-soaked in water for 18 h and treated with 0.1%, 0.3%, 0.5%, 1%, 1.5%, and 2% (v/v) of EMS (Sigma-Aldrich) in phosphate buffer. The treated seeds were incubated for 2 h, 4 h, 6 h, and 8 h at room temperature with gentle shaking, while untreated seeds were used as a control. Treated seeds were thoroughly washed, and a germination test was carried out on pre-wet filter paper placed in Petri dishes, incubated at ± 25 °C in the dark along with untreated seeds. The genotoxicity of EMS was assessed by analyzing the seed germination (%), seedling height (mm), and seedling vigor index on the 10^th^ day. The seedling vigor index (SVI) was calculated as suggested by [53]. Probit analysis was carried out to determine the concentration of EMS by assessing the relationship between the concentration and duration of EMS exposure. LD 50 value was used as an indicator in deciding the dose rate that causes maximum beneficial mutations with minimum lethality.

### Generation of mutant population

To mutagenize the seeds, 1.5% v/v EMS was applied for 4 h. The mutagenized seeds were transferred to 72-hole nursery trays filled with commercial-grade peat moss (BVB-Bio Flora, Singapore) and grown in indoor condition. The seedlings were transplanted individually into 2.5 L capacity plastic pots with 100% peat moss on the 17^th^ day after sowing (DAS). The plants were watered with N:P: K-15:15:15 + TE, a slow-release fertilizer (Bio Flora, Singapore) at weekly intervals. The internal growing condition was maintained at 22 °C with 65% relative humidity. M1 plants were manually pollinated, and seeds were harvested when silique became yellow. The mature seeds of each mutant plant were collected, air-dried, packed, labeled, and stored in a freezer at -20 °C [54].

### Visual selection of M2 phenotype with reduced elongation

**Fig. 1:**
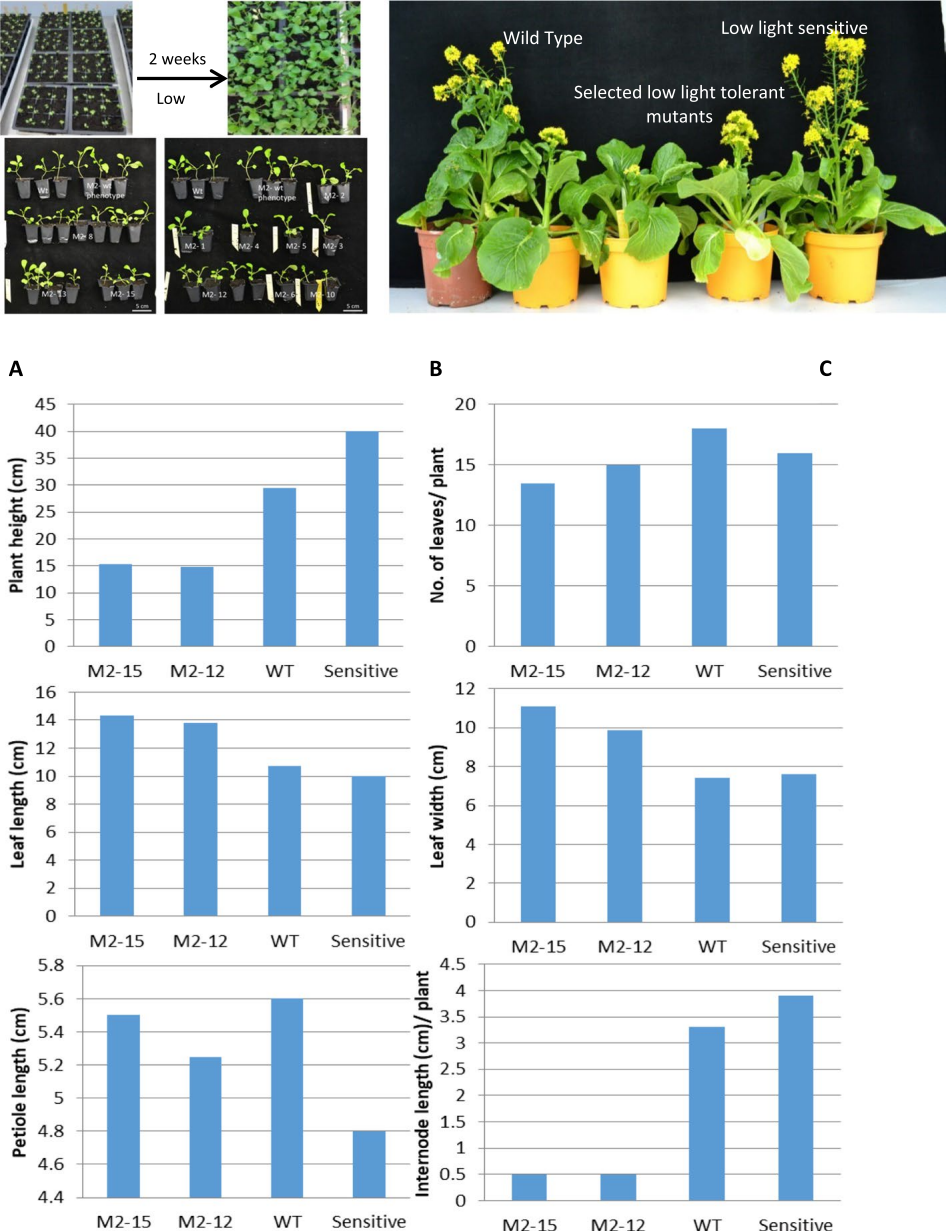
Screening and selection of M2 Choy Sum mutants. **A** screening and selection of M2 mutants under a low R: FR ratio (0.5), **B** Selected low light tolerant mutants with reduced plant height, **C** morphometric traits of control (wild type, [WT]/ untreated) plants and selected M2 mutants, the data were gathered from individual plants which were not replicated

A total of 3600 M2 seeds were screened in batches under low light to identify shade-tolerant mutants. Each time, 432 seeds were germinated in 72 plug trays filled with peat moss and grown under full-spectrum white LED light consisting of long red to short blue wavelength at 100 µmol m^−2^ s^−1^ of photosynthetic active radiation (PAR) for 10 days under a 16/8 h light–dark cycle. The PAR value was measured using Quantum PAR/DLI Light Meter (Spectrum Technologies, USA). A constant day/night temperature was set up at 22 °C with 65% relative humidity. On the 10^th^ day, the M2 seedlings along with the wild type were placed in low light (R: FR = 0.5) for two weeks and screened for shade tolerance. The reduced shade avoidance response was determined by absence of etiolation and a reduction in seedling height [30]. Seedling height measured between 6.00 cm to 9.00 cm was used as a guideline for selecting the mutants. 154 low-light-tolerant mutants were selected and transplanted into 2.5 L plastic pots and allowed to grow under full-spectrum white light (180 µmol m^−2^ s^−1^) at 22 °C under a 16/8 h light–dark cycle with 55–60% relative humidity in the indoor condition. Five mutants, viz*.,* M2-12–1; M2-12–2; M2-15–1; M2-15–2; and M2-15–3, that showed reduced plant height till maturity (Fig. 1) were hand pollinated; seeds were collected separately, labeled, and stored.

### Morphological and physiological evaluation of M3 mutants

Two sets of experimental materials were prepared in six replications for the evaluation of morphological and physiological traits in isolated M3 mutants. In each set, 20 seeds per mutant were sown in nursery trays along with the wild type and grown for two weeks by following the growth conditions as in the M2 generation. After two weeks, one set of the seedlings was transplanted into a 2.5L plastic pot and grown under full-spectrum white LED light (180 µmol m^−2^ s^−1^) at 22 °C under a 16/8 h light–dark cycle with 55–60% relative humidity. N:P:K-15:15:15 + TE, a slow-release fertilizer from Bio-Flora Pte Ltd-Singapore, was applied at weekly intervals at 2 g/ pot, and pots were kept well-watered and grown to maturity. The other group was placed in a 95% shade made of poly cloth [30] for a week before being reintroduced to full-spectrum white light. Leaf samples were collected at the end of the experiments (38 days old) and various morphological, physiological, and biochemical changes were evaluated. The plant growth responses of mutants to light and shade were compared with those of the wild type.

### Measurement of plant growth

Growth information on plant height (cm), number of leaves, leaf area, petiole length (cm), shoot fresh (FW, g) and dry weight (DW, g), leaf water content (%), and leaf yield (g) were recorded for both light and shade treated M3 plants at the time of harvest. Plant height was determined by measuring the maximal length from the apical to the base of the stem above the peat moss surface using a ruler. Leaf area was calculated by multiplying the maximum leaf length and leaf width. DW was determined after measuring the fresh weight in a precision balance (Mettler-Toledo, Switzerland) by drying the leaves in a hot-air oven at 65 °C for 3 days to measure the weight in grams per plant. Six uniform plants were sampled for estimation of chlorophyll content, enzymatic (total phenols, flavonoids, ferric reducing power, and proline content), and non-enzymatic antioxidant activities (SOD, POX, and CAT).

### Sample preparation for biochemical analysis

The fresh leaves were crushed into fine powder in a pestle and mortar using liquid nitrogen, and the samples were freeze-dried (Buchi Lyovapor L-200, Germany). About 100 mg of freeze-dried powdered leaf samples were extracted with 15 ml of 70% ethanol at room temperature, and the extract was left in an orbital laboratory shaker (Eppendorf Thermomixer F series, Germany) at 170 rpm for 24 h, then centrifuged (Sartorius-SIGMA 4K15 model, Mexico) on 4000 g at 4 °C for 10 min [55]. The filtrate was used to assay chlorophyll, carotenoids, phenols, and flavonoids.

### Estimation of chlorophyll content

Total chlorophyll content was determined by following the method described by Lichtenthaler [56]. About 1 mL of each plant extract was aliquoted into a microplate, and absorbance readings were measured at 470 nm, 649 nm, and 664 nm using a Tecan-Spark® Multimode Microplate Reader (Austria). The chlorophyll a, chlorophyll b, total chlorophyll, and carotenoid contents of mutants as well as wild type were calculated by the following equations and expressed in mg/g FW.

$$Chlorophyll_a=\;13\;.\;36A_{665}\;-\;5\;.\;19A_{649}$$

$$Chlorophyll_b=27.43A_{649}-8.12A_{665}$$

$$TotalChlorophyll=5.24A_{665}\;+\;22.24A_{649}$$

$$Carotenoid\;content=\frac{{1000}_{470}-2.13 C_a-97.{64} C_b}{209}$$

### Estimation of leaf nitrate content

About 1 g fresh leaves were homogenized in 0.1 M phosphate buffer using pestle and mortar to have a uniform distribution of nitrate content in the buffer, and leaf nitrate content was estimated using a multiparameter photometer (Hanna Instruments, model: HI83300, USA) by adopting the manufacturers' instructions. In brief, the cadmium reduction method was used to measure the nitrate content in the samples at a wavelength of 525 nm. The nitrate reagent (HI93728-0, Hanna Instrument, USA) was added to 10 mL of leaf sample extract and shaken for one minute. The sample was left to stand for 4 min and 30 s to display the reading of nitrate concentration (mg/L).

### Sample preparation and estimation of antioxidant assays

Enzymatic antioxidant assays were determined by following the method of Li et al., [57]. Fresh leaves (500 mg) were homogenized with 4.5 mL of 0.1 M phosphate buffer and incubated on ice for 10 min. The samples were centrifuged at 5000 g for 10 min at 4 °C. The supernatant was used to measure superoxide dismutase (SOD), catalase (CAT), and peroxidase (POD) activities using a respective assay kit (Sigma Aldrich, Germany) according to the manufacturer’s instructions.

### Analysis of free proline

Proline content was estimated by the modified procedure of Bates et al., [58]. Approximately 0.1 g of fresh leaf samples were homogenized using 5 mL of 3% aqueous sulfosalicylic acid by pestle and mortar. For 10 min, the homogenate was centrifuged at 12,000 g. 1 mL of the extract was used in a reaction with 1 mL of acid ninhydrin and 1 mL of glacial acetic acid and boiled at 100 °C for 1 h. The reaction was terminated rapidly on ice, and the resulting solution was extracted with 2 mL of toluene, mixed thoroughly, and left at room temperature for 30 min until the separation of the phases. 1 mL of the aqueous layer of the phase was placed on a microplate and the absorbance was measured at 520 nm. L-Proline (0.2, 0.4, 0.6, 0.8, 1.0, 1.2 µg/ml) was used as a standard and the equation was Y = 0.065x—0.006 (R^2^ = 0.9665). Where Y is the amount of free proline and x is the concentration of the samples. Proline concentration was estimated using the calibration curve and expressed as μg g − 1 FW.

### Determination of total phenol content

Total polyphenolic content was determined using the Folin-Ciocalteu reagent as described by Ahmad et al. [59] with some modifications for a 96-microplate method. 100 μL of the ethanolic extract was mixed with 500 μL of Folin-Ciocalteu reagent (fivefold dilution) and left in a shaker (Eppendorf Thermomixer F series, Germany) for 1 min at 30 rpm. After 4 min, 400 μL of sodium carbonate (75 g/L) was added to the mixture and left in the shaker for 1 min at 30 rpm. After 2 h at room temperature, the absorbance at 765 nm was measured with a Tecan-Spark® Multimode Microplate Reader (Austria). A calibration curve was prepared using gallic acid as a standard (20, 40, 60, 80, 100 µg/ml), and the equation was Y = 0.0085x + 0.0367 with R^2^ = 0.9967). The total phenolic content was calculated as gallic acid equivalents per gram of extract (mg GAE/g extract).

### Determination of total flavonoid content

Total flavonoid content was measured with the aluminium chloride colorimetric assay adapted from Chatattikun et al. [60] with slight modification. 100 µl of extracts was added to 20 µl of 10% aluminium chloride solution, followed by 300 µl of 96% ethanol. 20 µl of 1 M potassium acetate was added to the mixture in a 96-well plate and incubated for 30 min at room temperature, protected from light. 96% ethanol was used as a reagent blank, and the absorbance was measured at 415 nm with a microplate reader (Tecan-Spark® Multimode, Austria). Using quercetin as a standard (20, 40, 60, 80, 100 µg/ ml), a calibration curve was prepared, and the equation was Y = 0.0005x—0.0063 with an R^2^ = 0.99. Total flavonoid contents were expressed as mg of Quercetin Equivalents (QE) per g of plant extract.

### Determination of ferric-reducing power assay

Reducing power was determined by the method of Patel et al. [61]. 2.5 mL of the ethanolic extract was mixed with 1 mL of 0.2 M phosphate buffer and 1 mL of 1% potassium ferricyanide. The reaction mixture was incubated at 50 °C in a water bath for 20 min and cooled rapidly. 2.5 ml of 10% trichloroacetic acid was added to stop the reaction, then centrifuged at 4000 g for 10 min. 2.5 mL of the supernatant was mixed with 2.5 mL of distilled water and a freshly prepared 0.5 ml of (0.1%) ferric chloride solution and left to stand for 10 min. Absorbance was measured at 593 nm with a microplate reader (Tecan-Spark® Multimode, Austria), and the ferric-reducing power of leaf samples was determined using an ascorbic acid standard curve.

### Evaluation of stress tolerance indices

Leaf yield measurement was used to estimate the following stress tolerant indices.

- ○ Relative stress Index (RSI) = (ys/yp)/Ӯs/Ӯp)
- ○ Tolerance (TOL) = *y*p − *ys* [45]
- ○ Geometric mean productivity (GMP) = √((*y*p) (*ys*)) [47]
- ○ Yield stability index (YSI) = *ys*/*y*p [48, 49]
- ○ Stress resistance index (SRI) = (*ys*. (*ys*/*y*p))/Ӯ*s*
- ○ Abiotic tolerance index (ATI) = (*y*p − *ys*)/(Ӯp/Ӯ*s*) x (yp x ys) 0.5 [50]
- ○ Stress susceptibility index (SSI) = $(1-\left({y}_{s}/yp\right))/(1-\left(y\overline{s }/Y\overline{p }\right))$ [46]
- ○ Stress tolerance index (STI) = $\left({y}_{P} . {y}_{s}\right)/{y}_{P}^{2}$ [47]

where ys and yp are the leaf yields under shade and light of a mutant, Ӯs and Ӯp are average leaf yields of all mutants under shade and light conditions, respectively.

### PCR–RFLP analysis for mutant confirmation

#### DNA extraction

Total genomic DNA was extracted from young leaves by following Qiagen DNA mini kit (QIAamp) manual instructions. The Nanodrop 2000c UV/IV Spectrophotometer (ThermoScientific, USA) was used to assess the DNA quality for three samples (WT, M3-15–1, and M3-12–2). A 1% agarose gel electrophoresis was performed to confirm the purity of DNA.

### DNA Amplification and digestion of restriction enzymes

Phytochrome sequence information obtained from the GenBank database for Brassica napus (phytochrome A and C) transcript variants and *Brassica rapa* (phytochrome B) were used as a reference sequence to design the primer for PCR amplification. The details of the primers are presented in Table 1. The PCR reactions were conducted in a 50 µl mixture consisting of 25.0 μL 2 × iproof HF master mix (Bio-Rad) 25.0 μl, 4 μl each of primers (10 mM), and 4 μl DNA template. The PCR amplification was performed in the BioRad T100 Thermal Cycler (Bio-Rad Laboratories, Inc., USA) the conditions were as follows: initial denaturation at 95 °C for 2 min; followed by 35 cycles of denaturation at 95 °C for 15 s, annealing at 55 °C for 30 s, and extension at 72 °C for 40 s; and a final extension at 72 °C for 10 min. The PCR products were purified by QIAquick Gel Extraction Kit (Merck) and purified PCR products were used for DNA sequencing using an ABI BigDye Terminator Cycle sequencing kit v3.1 (1^st^ BASE, Singapore) according to the manufacturer’s instructions. Sequence alignment was performed using Clustal Omega tool (EMBL-EBI).

**Table 1:** Phytochrome primers used for PCR amplification

S.no	Primer	Direction	Sequence (5' → '3')	Size (bp)
1	PhyA	Forward (F)	ATCATTGCACAGACCACCGT	1190
		Reverse (R)	CATCGCGCATCAGCATATCG	
2	PhyB	Forward (F)	CGACTGGTTTAAGCACGGATAG	456
		Reverse (R)	CCAGCATCCACAGCGAATATAG	
3	PhyC	Forward (F)	CCGCCGTTTGTGGATAATAGAG	834
		Reverse (R)	GAACCTGTAGAGCGTATTGGAG	
4	PAR1	Forward (F)	TCTCTCTCTCTCTCACACACAC	250
		Reverse (R)	GCCGTTCCTCCGGGAATAAT	
5	PAR2	Forward (F)	GACGCGCTGTTTGAAGAGAC	255
		Reverse (R)	GCAAAAGAGCCAGCCACAAT	

The amplified PCR products were digested with restriction enzymes (*MspJI, FsPEI, MSeI, CviJI, HaeIII*, and *MnII*) directly, the 20 μL restriction reaction mixture consisted of 5 μL of the PCR product and 2 μL of the restriction enzyme in 2 μL of 10 × buffer and make up the volume of sterile free water. The reaction mixtures were incubated at 37 °C for 5–6 h and visualized using 3% agarose gel electrophoresis with 5 μL of sybr-safe DNA stain.

### Statistical analysis

Frequency distribution and the variance around the average of wild-type plants (the control) were performed for the M2 population because the data were gathered from individual plants that were not replicated. The Microsoft Excel program was used for the graphical presentation of data using standard error (± SE). M3 data in six replications (*n* = 6) were subjected to an analysis of variance (ANOVA) and Tukey’s HSD multiple mean comparisons (at the 0.05 significance level) after performing Levene’s test for homogeneity of variance using R software version 3.6.2. Principal component analysis (PCA) was performed using RStudio (RStudio Team, 2019) on 20 selected variables. The “prcomp” built-in R function with scale = TRUE and a ggbiplot was used. Eigenvalues > 1.0 were considered significant and the PCA biplot drawn using the first two principal components (PC1 and PC2) was overlaid with the hierarchical clusters. Estimations of various stress tolerance indices and the correlation coefficient of the indices were computed using Microsoft Excel.

## Results

### Development of a mutant population

**Fig. 2:**
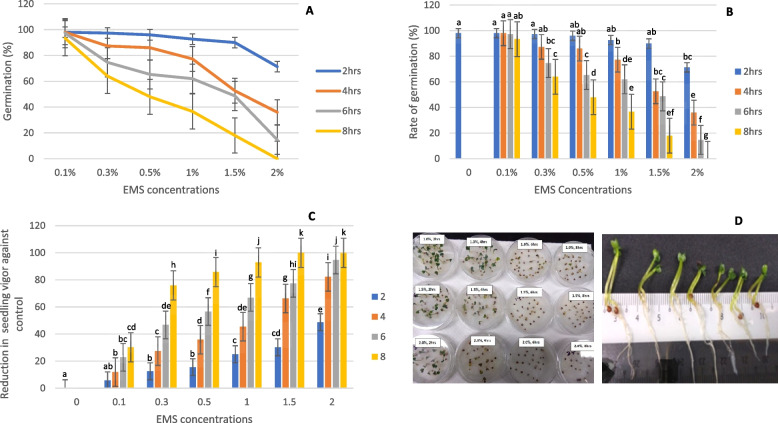
Effect of EMS concentration (%) on the germination rate of Choy Sum. **A** Germination percentage, **B** EMS effect on seed viability, **C** Percentage reduction in seedling vigor, **D**) Picture representing EMS effect on seedling growth. The results represent the mean from three independent biological replicates and the standard error (SE) are shown as vertical bars. The mean marked with different letters indicate a significant difference between treatments at p-value < 0.05, as determined with Tukey’s HSD test

The effectiveness of the EMS dose range (0.1%-2%) and treatment duration (2–8 h) were assessed based on seed viability and seedling vigor. These two parameters are obvious indicators to analyze the extent of tissue injury at the seedling stage. The rate of germination (%) reflects the measure of seed viability, increase in EMS concentration in the study (Fig. 2A) drastically affected the viability of the seed. 0.1–0.3% EMS showed a slight reduction in germination rate, and nearly 60% of the seeds were viable up to 8 h of incubation, whereas no seeds germinated with 2% EMS for 8 h. However, with 1.5% of EMS for 4 h, the seed viability was markedly reduced to 52%. Analysis of variance (Fig. 2B) showed a significant interaction between EMS concentrations and treatment durations (*P* < 0.05) for germination, indicating the relative importance of the duration of the treatment in deciding the optimal concentration. Probit analysis was carried out to examine the effect of dose–response on the survival of the seedling. The result presented in Table 2 showed that the LD50 (lethal dose 50) value of 1.5% EMS concentration at 4 h of treatment time caused maximum mutation with minimal damage to the plant. The result of the dose response was validated with the estimated seedling vigor index (SVI), which was calculated by dividing the percentage of viability by the seedling length. As with the rate of germination, EMS treatment also reduced seedling growth, and the retardation was intense at 1.5% and 2% dose levels treated for 6 h and 8 h (Fig. 2C). The higher dose severely inhibited root growth and consequently had a negative influence on seedling length (Fig. 2D). The estimate of SVI showed that 1.5% EMS for 4 h reduced the seedling vitality by 66% compared to the control. Based on the result, it was apparent that nearly 44% of mutagenized seedlings were able to thrive well and capable of producing seeds; hence, the dose rate of 1.5% EMS concentration at 4 h was fixed to generate a mutant population.

**Table 2:** Probit analysis to determine the lethal dose (LD 50) of various EMS concentrations exposed at 2 h, 4 h 6 h, and 8 h based on mortality rate in Choy Sum

Corrected mortality	Concentration (%)	Log Dose	Observed mortality	Empirical Probits	LC_50_ (%) [Min -Max]	Slope	Intercept	Chi-Square (df = 4)
2 h	0	0.00	0	0	17.29 (5.74—52.03)	0.995	3.768	0.04
	0.1	-1.00	2	2.95				
	0.3	-0.52	3	3.12				
	0.5	-0.30	4	3.25				
	1	0.00	8	0.00				
	1.5	0.18	10	3.72				
	2	0.30	29	4.45				
4 h	0	0.00	0	0.00	**1.55** (0.96—2.51)	1.759	4.663	0.678*
	0.1	-1.00	2	2.95				
	0.3	-0.52	13	3.87				
	0.5	-0.30	14	3.92				
	1	0.00	33	0.00				
	1.5	0.18	48	4.95				
	2	0.30	64	5.36				
6 h	0	0.00	0	0.00	0.97 (0.62—1.50)	1.86	5.070	0.164*
	0.1	-1.00	3	3.12				
	0.3	-0.52	26	4.36				
	0.5	-0.30	35	4.61				
	1	0.00	38	0.00				
	1.5	0.18	52	5.05				
	2	0.30	86	6.08				
8 h	0	0.00	0	0.00	0.54 (0.36—0.84)	1.93	5.533	0.766*
	0.1	-1.00	7	3.52				
	0.3	-0.52	36	4.64				
	0.5	-0.30	52	5.05				
	1	0.00	64	0.00				
	1.5	0.18	82	5.92				
	2	0.00	100	0.00				

*Significant difference at *P* < 0.05

### Evaluation of M3 mutants for shade tolerance

#### Morphological characteristics

**Fig. 3:**
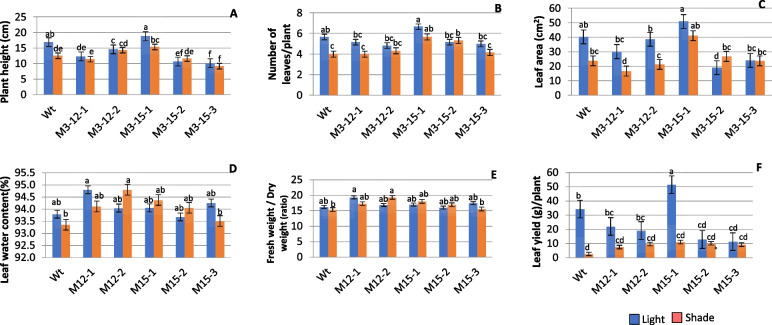
Morphological measures of selected M3 Choy Sum Mutants grown under light and shade. **A** plant height (cm), **B** the number of leaves/plants, **C** leaf area (cm^2^), **D**, leaf water content (%), **E**) the ratio of fresh and dry weight and **F**) leaf yield (g). Data are the mean of six replicates and SEs are shown as vertical bars. The mean marked with different letters indicate significant differences between treatments at *p*-value < 0.05, as determined with Tukey’s HSD test

The five dwarf Choy sum mutants tested in the shade showed significant (P < 0.05) morphological differences with the unshaded control (WT) at the vegetative stage (Fig. 3). The mutant M3-15–1 recorded a higher value for plant height (19%), more leaves (6.7%), greater leaf area (51%), and leaf yield (51%), compared to wild type in light conditions. Shade significantly suppressed the growth; the impact of shade stress was stronger in control and accounted for reductions in plant height (26%), the number of leaves per plant (29%), leaf area (41%), and leaf yield (94.4%). However, the growth of M3-15–1, M3-12–2, and M3-15–3 significantly improved after short exposure to shade.

### Physiological and biochemical analysis

#### Photosynthetic pigment and leaf nitrate content

**Fig. 4:**
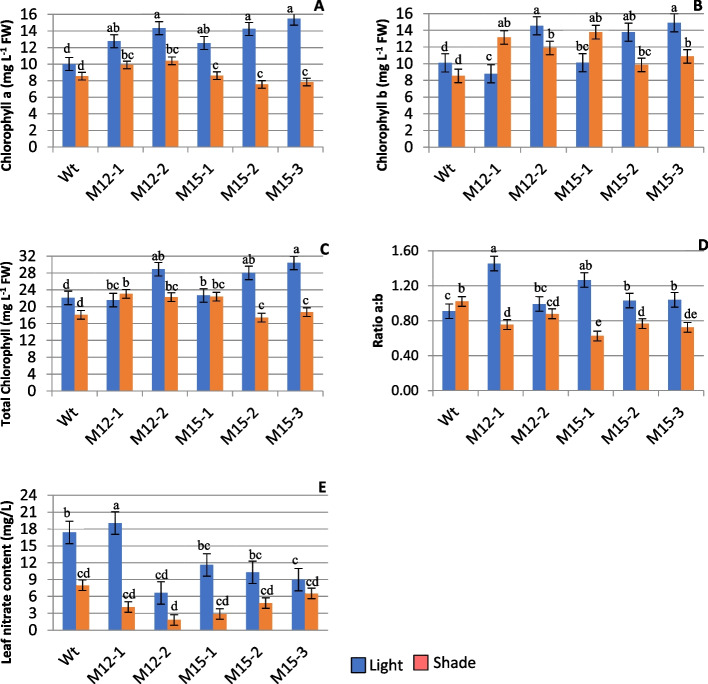
The change of chlorophyll content and leaf nitrate levels under light and shade. Five Choy Sum mutants and wild-type plants were used to quantify chlorophyll-a (**A**), chlorophyll-b (**B**), total chlorophyll (**C**), the ratio of chlorophyll a/b (**D**), and leaf nitrate content (**E**). Bars represent mean ± standard error (SEn = 6). The mean marked with different letters indicate significant differences between treatments *p* < 0.05, as determined with HSD test

The photosynthetic pigment was investigated in both light and shade conditions. The difference in light intensity significantly (P < 0.05) affected the pigment content (Fig. 4A-D). Chlorophyll-a was higher in light for all the mutants than the control. Shade decreased chlorophyll-a, and the difference between shaded control and mutants was significant. This reduction was high in M3-15–2 (47%) and M3-15–3 (49%) while M3-12–2 showed an increase (22%) in chlorophyll-a (Fig. 4A). Chlorophyll-b displayed a varying trend in light and shade; the overlapping of variations in chlorophyll-b failed to record a significant difference between light and shade. However, the chlorophyll-b content was higher than chlorophyll-a in shade, and a significant difference was observed between mutants and controls (Fig. 4B). The total chlorophyll content of M3-12–2, M3-15–2, and M3-15–3 significantly increased in the light except for M3-12–1, while M3-12–1 and 15–1 recorded significantly higher content in shade (Fig. 4C). The ratio of chlorophyll a/b was significantly higher in light than in shade; M3-12–1 and M3-15–1 had a higher ratio than the control in light (Fig. 4D). However, M3-15–2 (15%) and M3-15–3 (27%) showed a comparatively lower ratio in the shade.

Shade drastically reduced the leaf nitrate content (Fig. 4E) in the three M3 mutants, viz*.,* M3-12–1, M3-12–2, and M3-15–1. Assessing the percentage of leaf nitrate content reduction, the highest reduction was in M3-12–1 (78.32%), followed by M3-15–1 (75.30%), and M3-12–2 (72.56%), but an opposite trend was observed for M3-15–3 (27.09%) in the low light treatment.

### Antioxidant activities

**Fig. 5:**
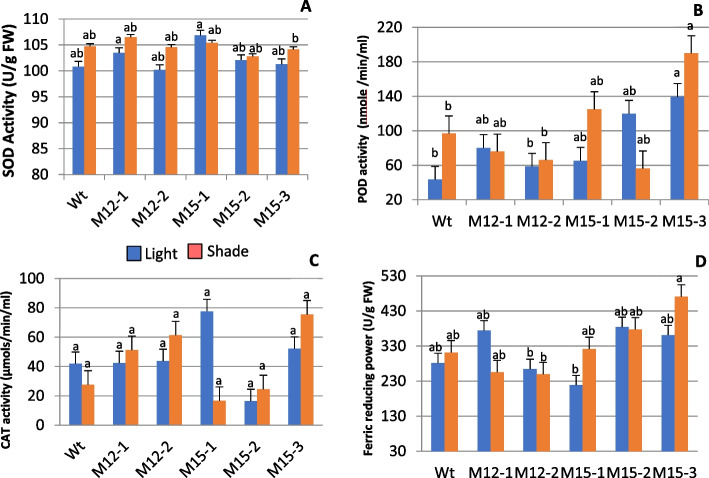
Effect of varying light on enzymatic antioxidants such as SOD activity (**A**), POD activity (**B**), CAT activity (**C**), and ferric reducing power (**D**) in the leaves of five mutants and wild type Choy Sum. Data are the mean of six replicates and the SE (*n* = 6) are shown as vertical bars. The mean marked with different letters indicate significant differences between treatments at *p*-value < 0.05, as determined with Tukey’s HSD test

The enzymatic antioxidant activities of SOD, CAT, and POD under light and shade were analyzed, and the results are presented in Fig. 5 (A-D). Shade significantly induced SOD activity (*P* < 0.05) in M3-12–1 and M3-15–3 when compared to light, whereas M3-15–1 had lower SOD activity. POD activity showed no significant changes between light and shade, yet different mutants displayed differences in activity. POD was higher in M3-15–1 and M3-15–3, while significantly lower activity was observed in M3-15–2 compared to the control. CAD activity showed no significant changes between light and shade or among mutant lines. The ferric-reducing antioxidant power assay (FRAP) had no significant difference with the light intensity, but the value of FRAP was significantly high in M3-15–3 while it was low in M3-12–2 and M3-15–1.

**Fig. 6:**
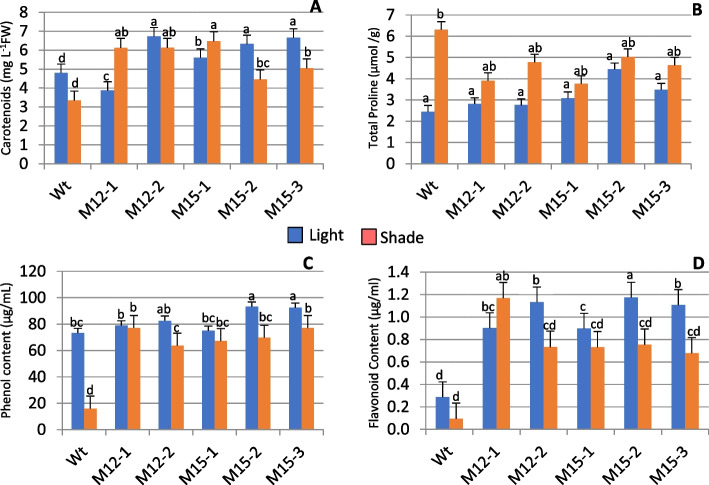
The change in non-enzymatic antioxidant effect of Choy Sum mutants and wild type grown under light and shade. Five Choy sum mutant and wild-type plants were used to quantify carotenoid content (**A**), Total proline content (**B**), Phenol content (**C**), and Flavonoid content (**D**). Bars represent mean ± SE (*n* = 6). The mean marked with different letters indicate significant differences between treatments *p* < 0.05, as determined with Tukey’s HSD test

Non-enzymatic antioxidants like carotenoids, proline, phenols, and flavonoids were also analyzed in response to changes in light (Fig. 6A-D). Carotenoids caused no significant difference between light and shade, but varying responses to carotenoids were exhibited among the mutants (*P* < 0.05). Light-induced higher carotenoids in M3-12–2, followed by M3-15–2 and M3-15–3. On the contrary, shade increases carotenoid content in M3-12–1 and M3-15–1. Proline content was considerably influenced by shade. When compared to the shaded control, the level of proline was lower for the mutants. However, no significant difference was observed. Shade notably suppressed total phenolic content, and the reduction was substantial in control. M3-12–1 and M3-15–3 registered higher total phenolic content in light. Like phenol, total flavonoid content was significantly higher in light than in shade. Compared to the control, M3-12–2, M3-15–2, and M3-15–3 had higher flavonoids in light, while the flavonoid content of M3-12–1 was high in the shade.

### Principal component analysis (PCA)

**Fig. 7:**
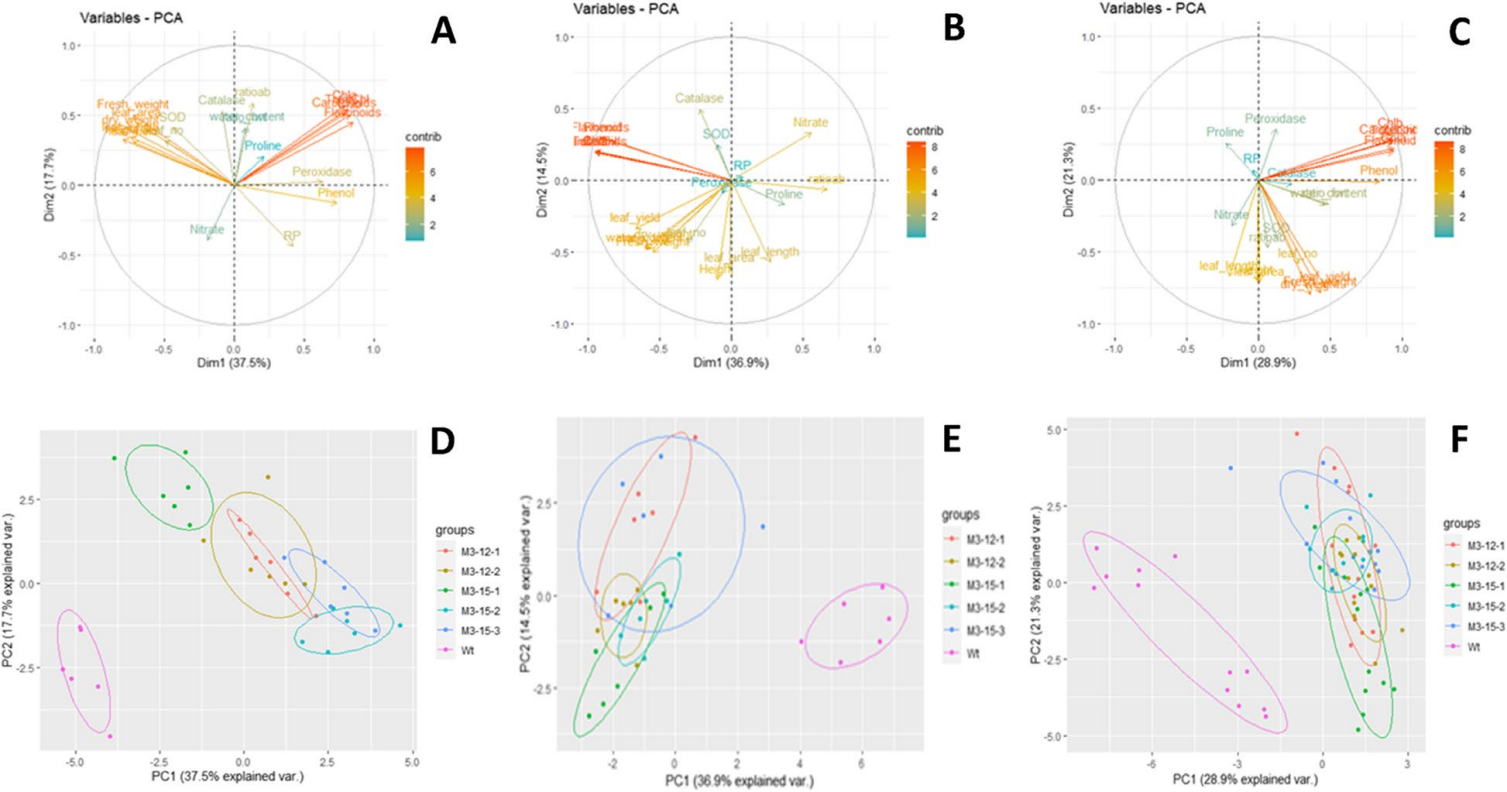
(**A**-**F**). Principal component analysis biplot of five M3 Choy Sum mutants and control (WT) tested under light, shade, and combined condition. The twenty-five morphological and physiological parameters allow to separate 5 mutants: Factor loading showing contribution of traits to PC1 and PC2 for: light (**A**), shade (**B**), combination (**C**). PCA biplot showing grouping of variables; light (**D**), shade (**E**) and combined (**F**). Nonoverlapping circles are statistically significant (*p* < 0.05). The further the distances between circles, the stronger the differences between the groups

Principal component analysis was done to identify the grouping pattern based on morphological such as plant height, leaf number, leaf area, leaf yield as well as physio-chemical parameters like leaf water content, chlorophyll pigments, enzymatic and non-enzymatic antioxidants among the mutant and control (Wt) grown under shade, and the relationships are graphically displayed in Fig. 7 (A-F). The first two principal components accounted for 37.5% (PC1) and 17.7% (PC2) of the genetic variance (Fig. 7A) in light conditions. Five principal components with eigenvalues greater than 1 explained 79.63% of the variation in the data. PC1 accounted for the contrast among the choy sum mutants for ascorbate peroxidase, total phenolic content, and peroxidases with their positive coefficients, while leaf fresh and dry weight, leaf area, number of leaves per plant, and SOD with their negative coefficients. Similarly, PC2 represented the variation for chlorophyll-a, chlorophyll-b, total chlorophyll, carotenoids, and total flavonoids with a positive coefficient and leaf nitrate content with a negative coefficient. The most representative variables were flavonoids, chlorophyll, and carotenoids. PCA showed (Fig. 7D) distinct clustering patterns for the five M3 mutants and oriented away from the control (Wt). However, a slight overlap observed for M3-12–1, M312-2, M3-15–2, and M3-15–3 indicated that these mutants most likely have some similarity.

As illustrated in Fig. 7B, the variable contribution for shade was 36.9% (PC1) and 14.5% (PC2), respectively. Six PCs had eigenvalues of > 1 and contributed 83.89% of the cumulative variability. PCA1 was represented by plant height, leaf area, and the number of leaves with a positive coefficient and catalase with a negative coefficient. While PC2 displayed the variation for leaf nitrate content and chlorophyll a/b ratio with a positive coefficient, the negative coefficient was represented by leaf yield, leaf fresh, and dry weight. The biplot (Fig. 7E) indicated that the five mutants were discrete from the control but heavily overlapped among themselves.

The combination of both light and shade conditions had the factor of PC1 explain about 28.9% of variables and PC2 about 21.3% (Fig. 7C). Six PCs had eigenvalues > 1 with 78.05% of cumulative variability. PC1 was represented by leaf yield-contributing traits, whereas PC2 was with proline. The PCA plot of mutants illustrated (Fig. 7F) showed that heavily overlapped groups of mutants were oriented away from the control.

### Stress tolerance indices

**Fig. 8:**
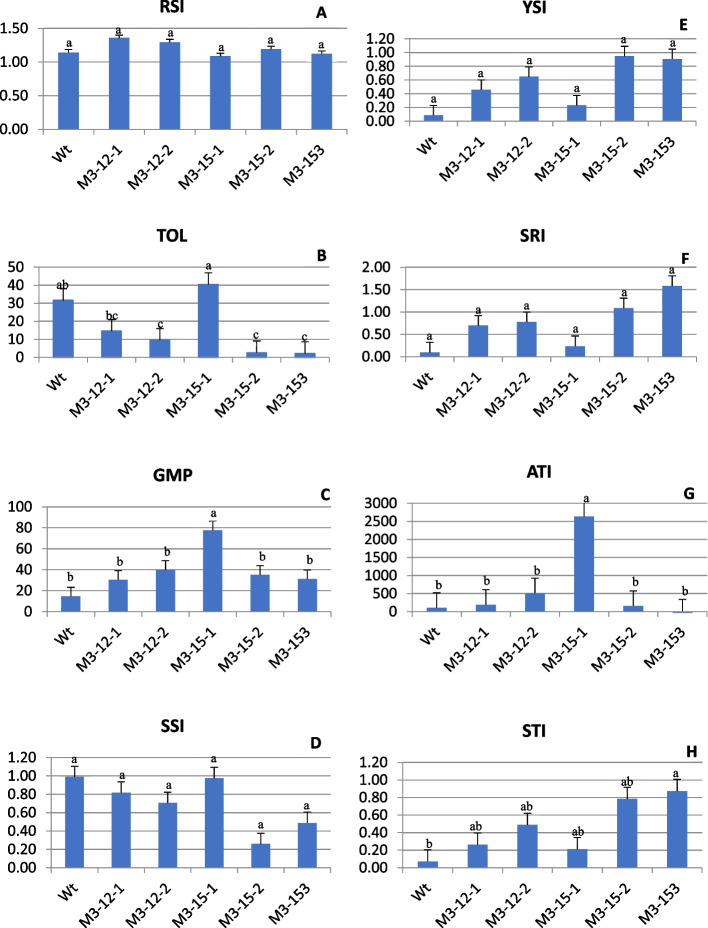
(**A**-**H**) Graphs of different stress response indices of five M3 mutant and wild type Choy Sum: RSI (**a**), TOL (**b**), GMP (**c**), SSI (**d**), YSI (**e**), SRI (**f**), ATI (**g**), and STI (**h**). Data are the mean of six replicates and SEs are shown as vertical bars. The mean marked with different letters indicate significant differences between treatments at *p*-value < 0.05, as determined with Tukey’s HSD test

Various stress tolerance indices were computed, and the result is illustrated in Fig. 8 (A-H) and Supplementary Table 1. Among the indices, TOL, GMP, ATI, and STI recorded significant (*P* < 0.05) differences between mutant and control. This demonstrated that the variations was adequate to choose mutants with increased resistance to shade stress. M3-15–1 showed higher significant variations for TOL, GMP ATI, and Low SSI, whereas M3-15–3 had higher values for STI.

In the present study, a significant positive correlation was found for TOL, MP, and ATI, with leaf yield under light conditions (Table 3). While indices such as RSI, STI, GMP, YSI, and SRI were positively associated with leaf yield under shade had a negative association with TOL and SSI. Selection based on higher values of STI, GMP, YSI, and SRI and lower values of ATI and SSI were considered appropriate indices to select shade tolerance. The three mutants M3-12–2, M3-15–2 and M3-15–3 had similar indices mentioned above in shade condition.

**Table 3:** Pearson correlation coefficient (r) between different stress indices of EMS induced five M3 Choy Sum mutants. SSI, stress susceptibility index; RSI, relative stress index; TOL, tolerance index; MP, mean productivity; STI, stress tolerance index; GMP, geometric mean productivity; YSI, yield stability index; SRI, stress response index; ATI, abiotic tolerance index estimated from leaf yield under light and shade condition

	*SSI*	*RSI*	*TOL*	*MP*	*STI*	*GMP*	*YSI*	*SRI*	*ATI*	*Leaf Yield (L)*
SSI	1.000									
RSI	-0.749^a^	1.000								
TOL	0.383^a^	-0.548^a^	1.000							
MP	-0.009^ ns^	-0.149^ ns^	0.811^a^	1.000						
STI	-0.396^a^	0.251^ ns^	-0.453^a^	-0.034^ ns^	1.000					
GMP	-0.325^a^	0.360^a^	0.135^ ns^	0.668^a^	0.440^a^	1.000				
YSI	-0.843^a^	0.749^a^	-0.743^a^	-0.323^a^	0.589^a^	0.270^ ns^	1.000			
SRI	-0.862^a^	0.710^a^	-0.571^a^	-0.114^ ns^	0.653^a^	0.377^a^	0.889^a^	1.000		
ATI	0.288^ ns^	-0.340^a^	0.727^a^	0.792^a^	-0.146^ ns^	0.539^a^	-0.423^a^	-0.388^a^	1.000	
Leaf Yield (L)	0.203^ ns^	-0.373^a^	0.955^a^	0.948^a^	-0.263^ ns^	0.413^a^	-0.567^a^	-0.367^a^	0.797^a^	1.000
Leaf Yield (S)	-0.653^a^	0.681^a^	-0.401^a^	0.211^ ns^	0.703^a^	0.820^a^	0.734^a^	0.775^a^	0.025^ ns^	-0.110^ ns^

^a^and ^ns^: significant at 5% level and not significant

**Fig. 9:**
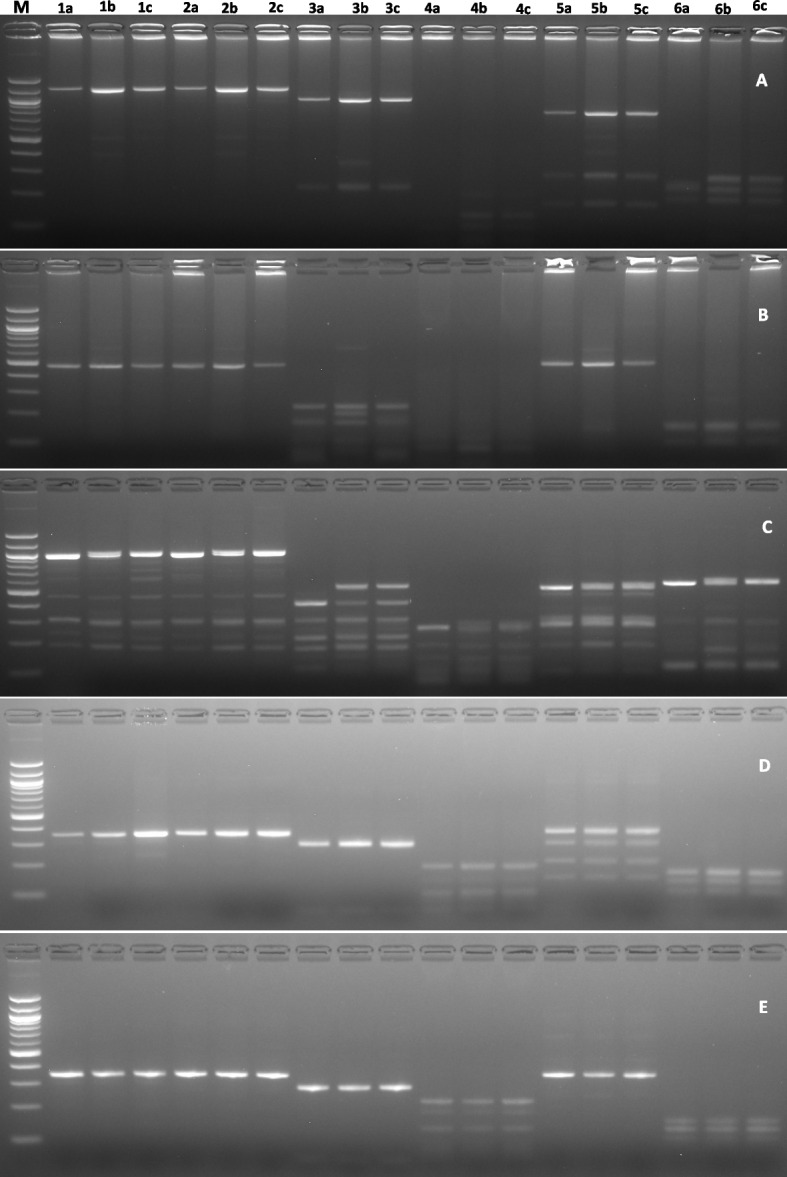
PCR–RFLP amplification of Choy sum wild type (a), M3 12–2 (b), and M3-15–1 after digesting with restriction enzymes. **A** Gel electrophoresis results of PCR‐RFLP for PHYA, **B** PHYB, **C** PHYC, **D** PAR1 and **E** PAR2. Lanes 1a-1c digested with restriction enzyme MspJ1, Lanes 2a-2c with FsPE1, Lanes 3a-3c with Mse1, Lanes 4a-4c with CviK1, Lanes 5a-5c with Hae111 and Lanes 6a-6c with Mn11. M is 100 bp DNA ladder marker (New England Bio Labs, UK)

**Fig. 10:**
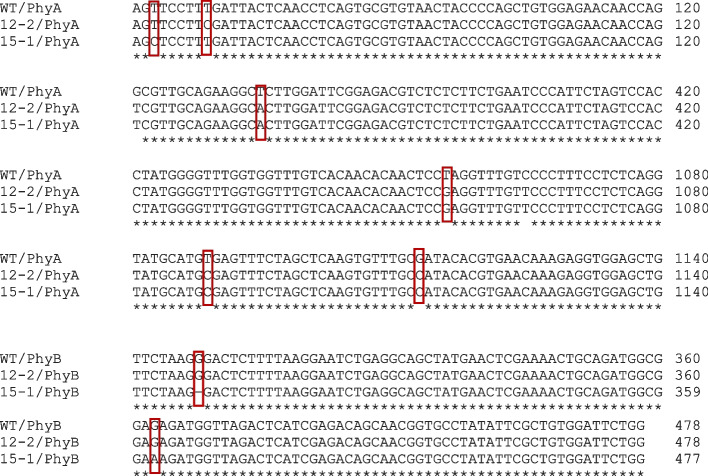
Clustal Omega multiple sequence alignment from the PCR-amplified regions of PHYA and PHYB DNA sequence

PCR–RFLP analysis was carried out to verify the genetic variations in two mutants M3-12–2, M3-15–1, and WT phytochrome gene, Fig. 9 illustrates the band sizes obtained by restriction digestion with *MspJI, FsPEI, MSeI, CviJI, HaeIII,* and *MnII*. The two mutants and wild type showed a similar pattern of restriction with the six enzymes for PHYA, PHYB, PAR 1, and PAR2. While PHYC displayed distinct band variations and was able to differentiate the mutants and the wild types. PCR amplified with PHYC digested with *MspJ1* produced seven fragments M3-15–1. *FsPE1* produced four fragments in M3-12–2 and M3-15–1, *MSe1* produced five fragments in M3-12–2 and M3-15–1, *CviK1* produced five fragments in M3-12–2, *Hae111* produced four fragments in M3-12–2 and M3-15–1 and *Mn11* produced five fragments in M3-12–2 (Table 4). However, all the restriction enzymes invariably produced a lesser number of bands in the wild type. In the sequence presented in Fig. 10. showed nucleotide variation for PHYA and PHYB alone.

**Table 4:** Restriction enzymes and number of polymorphic resultant fragments from PCR–RFLP of Choy sum Wild type (WT), Mutants M3-12–2 and M3-15–1

	Number of fragments		
Restriction enzymes	WT	M3-12–2	M3-15–1
MspJ1	5	5	7
FspE1	3	4	4
Mse1	4	5	5
CviK1	3	4	3
Hae111	3	4	4
Mn11	3	5	4

## Discussion

The effectiveness of an EMS dose on Choy sum was assessed based on seed viability and seedling vigor index (SVI); these two components are considered as a means for potential seedling establishment under a wide range of environmental conditions [62]. In this study, we observed that the rate of germination and seedling growth was greatly affected by both an increase in concentration and the treatment duration of EMS (Fig. 2). The greater reduction in seed germination may be attributed to the inhibition of metabolic processes involved in hydrolysis, biosynthesis of macromolecules, respiration, and cell elongation during germination [63]. Studies have inferred that those differences in seed vigor manifest through varying effects on the rate of germination and seedling growth [64–66]. Sometimes a viable seed may be unable to continue its growth and development to complete the life cycle, so the vigor test was recommended as the most relevant practice to determine the seed quality. The test is commonly used to detect storage potential, mechanical damage, pest, and pathogen infestations in seeds [67, 68]. Recently, SVI has been used as a phytotoxicity index to evaluate the effectiveness of mutagens on seedling growth. Based on the assessment of seed viability and seedling vigor, 1.5% EMS at 4 h was optimal for generating mutant populations of Choy Sum. In the previous study, Stephenson et al. [69] used 0.3%–0.4% EMS at 16 h to induce variations in *Brassica rapa* subsp. *Trilocularis* (Yellow Sarson) seeds. Similarly, Yang et al. [70] reported that 0.5% EMS at 16 h was effective for turnip seeds to produce mutants. In another study, cabbage buds soaked in 0.03 to 0.1% EMS for 5 to 10 min produced more embryos and higher seedling rates [71]. Those studies indicated that a low dose may be required to produce a large mutant population, while for a smaller population, the mutation frequency needs to be high enough to effect desirable change. A mutant population of Choy sum was constructed using the optimized EMS dose rate, and the plants were manually pollinated to recover seeds to raise subsequent generations.

The majority of M2 seedlings tested under a low R: FR ratio displayed elongated hypocotyl, stem, petiole growth, and chlorotic leaves (Fig. 1). Light perception studies on plants specified that the plant perceives differences in light quality, and these changes trigger a set of responses known as shade avoidance syndrome (SAS), characterized by elongation of hypocotyl, stem, and petiole, apical dominance, early flowering, decreased leaf expansion, and decreased yield [5, 72, 73]. Li et al.’s [30] belief that de-etiolated plant traits under low light would be advantageous in enhancing shade tolerance led to the development of a new turfgrass cultivar through a two-step screening process in which three-leaf stage seedlings were initially screened to isolate dwarf mutants and mature plants were later screened under low light to identify shade tolerance. We followed the method suggested by Li et al. [30] for isolating putative shade-tolerant Choy sum mutants. Nearly 154 dwarf-mutant lines were identified, of which 5 exhibited putative shade tolerance, accounting for about 3.25% of all recovered dwarf-mutant lines.

We compared the effect of both light and shade on five M3 dwarf mutants based on several morphological, physiological, and biochemical adaptive responses for the suitability of CEA. Our result demonstrated that shade stress significantly suppressed growth traits; however, the mutants differed widely in the magnitude of response. M3-15–1 and M3-12–2 had higher measures for morphological parameters, viz*.*, plant height, leaf water content, and the ratio of fresh weight to dry weight, compared to the shaded control (Fig. 3). The phenotypic variation explained by shade conditions revealed differences among the mutants in adaptation to low light intensity, suggesting that selection based on these adaptive variations would be relevant in identifying shade-acclimated genotypes [74–77].

We analyzed the photosynthetic pigments to verify the adaptability of the mutants to shade, and the study showed that chlorophyll-b content was relatively higher than chlorophyll-a and resulted in a low chlorophyll a/b ratio (Fig. 4). M3-15–1 recorded the highest chlorophyll-b content in shade, followed by M3-12–2 and M3-12–1. An increase in the relative proportion of chlorophyll-b to chlorophyll-a indicated that plants accumulate more chlorophyll-b to increase the absorption of blue-violet light under reduced illumination to support plant growth [78–80]. Furthermore, studies have shown that chlorophyll-a is primarily associated with reaction center complexes, whereas chlorophyll-b is associated with the distal antennae of the light-harvesting chlorophyll protein complex (LHCII), and that chlorophyll-b changes reflect the changes in distal antennae size [81–83]. The ratio of chlorophyll a/b ranked higher in M3-12–1 and M3-15–1 in light, while M3-12–2 was higher in shade. The variation in chlorophyll a/b ratio displayed the difference in the composition and function of the light-harvesting complex in Choy Sum mutants. The measure of chlorophyll a/b under the shade provided an understanding of the photosynthetic regime and served as an indicator in the selection of tolerant mutants.

The ability of Choy Sum mutants to tolerate shading can be evaluated by analyzing antioxidative potential, as environmental stresses trigger both enzymatic and non-enzymatic antioxidant machinery to regulate ROS and ensure the survival of plants. In shade, M3-12–2 had higher values for SOD, CAT, and POD activity, suggesting that the lower R: FR condition induced an efficient ROS scavenging mechanism. Increased activity of enzymatic antioxidants in response to lower R: FR treatment was reported by Cao et al. [84] in tomatoes. While M3-15–1, which had higher leaf yield in shade, showed lower activity for SOD and CAT, indicating sensitivity to ROS scavenging. FRAP value varied with the mutants, and no significant variation was noticed between light and shade. M3-15–1 and M3-15–3 had high FRAP for shade. Ahmed et al. [85] also detected an increment of antioxidant capacity (FRAP) with increasing abiotic stress (salinity) in barley.

Carotenoids are primarily involved in pigmenting and protecting photosynthetic structures. They interact with free radicals as well as singlet oxygen in dissipating excess absorbed energy during stress conditions [86]. Shade generally reduced carotenoid content, which is consistent with the findings of Zhu et al. [87], who discovered that the carotenoid content of purple Pak-choi decreased under low light stress. However, the higher carotenoid content of M3-12–1 and M3-15–1 under shade (Fig. 6) contributed less damage to chlorophyll.

Proline is a well-established compatible osmolyte that displays a diverse role in response to a range of abiotic stresses [88], including osmotic balance, stabilization of subcellular structures, and scavenging free radicals [84]. Trovato et al. [89] pointed out that during abiotic stress, proline accumulates rapidly in the cells and degrades immediately once the stress is over. We examined the accumulation of this osmolyte in Choy Sum leaves. As expected, the amount of proline was significantly higher in shade than in light. Mounting evidence suggests that the accumulation of proline is positively associated with plant stress and has been identified as a key indicator of shade tolerance [88]. However, the study implicated that the higher accumulation of proline for control than the mutants in shade was due to a hypersensitive response to modification of plant development. The accumulation of a higher amount of proline was consistent with the findings of Liang et al. [90] and Kanawapee et al. [91]. They demonstrated that the sensitive cultivar tends to accumulate more proline than the tolerant and suggested that over-accumulation of proline was a symptom of injury.

Plant polyphenols such as phenols and flavonoids are good indicators to predict the extent of stress tolerance in plants [92]. Our study has shown that the level of polyphenols was comparatively lower in shade than in light, but M3-12–1 had higher phenol and total flavonoid content, which may be due to increased ROS induced by shade (Fig. 6), suggesting that enhanced production of these compounds under abiotic stresses is capable of scavenging free radicals by regulating the biosynthesis of secondary metabolites in order to protect the plants from adverse effects [93, 94].

Associations of morphological, physiological, and biochemical traits of mutants were analyzed using principal component analysis. Here we demonstrate that the traits such as leaf fresh and dry weight, leaf area, number of leaves per plant, ascorbate peroxidase, total phenolic content, peroxidases, SOD photosynthetic pigments viz. chlorophyll-a, chlorophyll-b, total chlorophyll, carotenoids, total flavonoids (Fig. 7A-F) had the highest variation in PCA. Furthermore, fresh weight, dry weight, leaf area, leaf yield, and antioxidants have contributed more towards cluster separation in light and shade. PCA revealed a clear distinction between mutant and control (Wt) based on their changes in morphological, physiological, and biochemical traits in response to shade. The result was well supported by the earlier findings [95–97] where PCA was used to differentiate the degree of tolerance/sensitivity to abiotic stress conditions.

Fernandez [47] suggested the use of different indices to select a genotype under both control and stress conditions. The indices TOL, and SSI was high in M315-1 indicating that the mutant was relatively susceptible to stress and can experience a greater yield reduction. Therefore, the selection of M3-15–1 would be appropriate only for under light conditions. Nouri et al. [98] suggested that TOL and SSI with smaller values had low-stress susceptibility and high yield stability. Such an index would be ideal for the selection of stress tolerance. Furthermore, Fernandez [47] proposed that selection for higher STI value reflects greater stress tolerance and higher yield in stress environment. MM3-15–2 and M3-15–3 had low TOS and SSI and a high STI value indicating that the mutants were less susceptible to shade and had high yield stability under low light conditions. However, the size of those mutants was too small. Individual plants weighed between 9 and 13 g in both shade and light conditions. Fischer and Maurer [46] specified that a value of SSI below 1.0 is regarded as tolerant to stress. M3-12–2 had an SSI value of 1.0 and moderately high values of GMP, STI, YSI, and SRI. Further, the correlation coefficient among different indices with leaf yield under stress showed a strong positive significant association with RSI, STI, GMP, YSI, and SRI. The result supports the findings of Thiry et al. [99] that GMP and STI indices showed a positive correlation with yield, which are suitable to identify genotypes with stress tolerance and high average yield.

Though PCR–RFLP showed no fragment variations for PHYA, PHYB, PAR1, and PAR2 except for PHYC, the nucleotide variations in the sequence of PHYA and PHYB enabled to differentiate between the wild type and the mutant strain.

## Conclusion

Comparison of an EMS-mutagenized population of M3 Choy sum with an untreated control population displayed wide variation for morphological, physiological, and biochemical adaptive responses to light and shade. An estimate of PCA displayed interrelationships among chlorophyll-a, total chlorophyll, chlorophyll-b, flavonoids, carotenoids, and plant height for light, while traits like flavonoids and phenols were associated with shade. Furthermore, PCA distinguished mutants from untreated controls based on the traits analyzed under light and shade. Stress tolerance indices such as RSI, STI, GMP, YSI, and SRI discriminated against the mutants for shade, and the study identified M3-12–2 as shade-tolerant. The mutant manifested moderate plant height, elevated levels of leaf water content, fresh and dry weight ratios, and antioxidants, along with lower estimates of chlorophyll, carotenoids, phenols, and flavonoids in shade. Our results demonstrated that mutation breeding can be used to generate heritable variation in Choy Sum, and various stress tolerance indices were highly resourceful in screening and selecting shade tolerance mutants.

### Supplementary Information

**Additional file 1.** 

## Data Availability

The datasets used and analysed during the study can be obtained from the corresponding author on reasonable request.
