# Supplementary material for: EMS-induced mutagenesis in Choy sum (Brassica chinensis var. parachinensis) and selection for low light tolerance using abiotic stress indices
Source: BMC Plant Biol. 2023 Nov 21;23:581. doi: 10.1186/s12870-023-04570-8 (PMC10662144; doi:10.1186/s12870-023-04570-8)
Supplement: Supplementary file 1 — Additional file 1. [file 12870_2023_4570_MOESM1_ESM.docx]

| Samples | WT | M3-12-1 | M3-12-2 | M3-15-1 | M3-15-2 | M3-15-3 |
| --- | --- | --- | --- | --- | --- | --- |
| SSI | 0.99 ± 0.02 | 0.82 ± 0.24 | 0.71 ± 0.34 | 0.98 ± 0.05 | 0.26 ± 1.20 | 0.49 ± 1.94 |
| RSI | 1.14 ± 0.23 | 1.36 ± 0.47 | 1.29 ± 0.34 | 1.09 ± 0.17 | 1.19 ± 0.31 | 1.12 ± 0.46 |
| TOL | 31.59 ± 5.11 | 14.56 ± 5.90 | 9.52 ± 5.59 | 40.39 ± 6.43 | 2.63 ± 2.84 | 2.16 ± 4.01 |
| MP | 18.38 ± 2.41 | 14.71 ± 1.18 | 14.3 ± 2.64 | 31.19 ± 3.07 | 11.43 ± 1.31 | 10.2 ± 2.38 |
| STI | 0.07 ± 0.01 | 0.26 ± 0.06 | 0.49 ± 0.15 | 0.21 ± 0.03 | 0.78 ± 0.18 | 0.87 ± 0.36 |
| GMP | 14.61 ± 2.04 | 30.44 ± 8.03 | 39.95 ± 10.59 | 77.7 ± 6.22 | 35.21 ± 6.37 | 31.21 ± 12.58 |
| YSI | 0.09 ± 0.02 | 0.46 ± 0.16 | 0.65 ± 0.17 | 0.23 ± 0.04 | 0.95 ± 0.25 | 0.91 ± 0.37 |
| SRI | 0.1 ± 0.03 | 0.7 ± 0.31 | 0.78 ± 0.29 | 0.24 ± 0.05 | 1.08 ± 0.34 | 1.58 ± 1.14 |
| ATI | 104.98 ± 22.27 | 189.78 ± 54.86 | 512.45 ± 402.41 | 2629.12 ± 768.13 | 158.93 ± 140.43 | 79.73 ± 33.11 |
| Yield L | 34.18 ± 4.95 | 21.99 ± 3.90 | 19.06 ± 4.95 | 51.38 ± 6.24 | 12.75 ± 2.18 | 11.28 ± 2.34 |
| Yield S | 2.58 ± 0.38 | 7.43 ± 2.22 | 9.54 ± 2.25 | 10.99 ± 0.72 | 10.12 ± 1.65 | 9.12 ± 3.73 |

Suppl. Table 1. Calculated stress tolerance indices of five M3 mutants and wild types for low light tolerance and selection of Choy sum mutants
